# A case report of acute testicular pain secondary to segmental testicular infarction

**DOI:** 10.1186/s12894-022-01006-7

**Published:** 2022-04-05

**Authors:** Hong-Liang Jin, Qi Ma, Jin Zhu, Ya-Chen Zang, Yi-Bin Zhou, Bo-Xin Xue, Dong-Rong Yang, Chuan-Yang Sun, Jie Gao, Li-Jun Xu, Bo Zhang

**Affiliations:** Department of Urology, The Second Affiliated Hospital of Soochow University, 1055 Sanxiang Rd, Suzhou, 215004 China

**Keywords:** Segmental testicular infarction, Testicular pain, Infarct blood flow, Case report

## Abstract

**Background:**

Segmental testicular infarction is a rare condition that often occurs in the upper pole of the left testicle and usually presents with acute onset of scrotal pain. Contrast-enhanced ultrasound and MR are essential for diagnosing and differentiating segmental testicular infarction in clinical practice, and conservative treatment can only be adopted after a definitive diagnosis. In the present case, after conservative treatment, the infarct volume was reduced, the blood flow around the infarct was increased, and blood flow signals appeared in the infarct. We performed a correlation analysis to investigate the causes of these changes.

**Case presentation:**

A 33-year-old male, without any specific disease history, was admitted to the hospital with a 5-day history of left testicular pain, and the imaging showed focal necrosis of the left testicle with hemorrhage. He was diagnosed with segmental testicular infarction after differentiating and excluding it from malignant tumors. Conservative medical treatment was given, and the symptoms of testicular pain were relieved after treatment. After discharge, regular reexamination at follow-ups showed that the infarct’s size was reduced, the blood flow around the infarct was increased, and blood flow signals appeared in the infarct.

**Conclusion:**

Conservative treatment has become the standard treatment currently adopted after confirming the diagnosis of segmental testicular infarction through contrast-enhanced ultrasound and MR. The blood flow changes in and around the focus of testicular infarction can be related to various factors. At present, relevant conclusions of the underlying mechanisms were mainly deduced from infarction studies of other related organs such as the heart and brain; thus, the specific pathological mechanism needs further experimental verification.

## Background

The incidence of segmental testicular infarction is extremely low. The cause is mainly idiopathic [1, 2], but it can also be related to blood hypercoagulability states (such as deficiency of antithrombin III or protein S), vasculitis, torsion, trauma, infection [3], and iatrogenic vascular injury. Later, polycythemia, intimal fibroplasia of the spermatic artery, and sickle cell disease were also discovered to be susceptible factors. Segmental testicular infarction mostly occurs in young men aged 20–40, and its typical clinical manifestation is acute scrotal pain, which needs to be differentiated from testicular tumors, testicular torsion, epididymitis, orchitis, etc. Ultrasonography can distinguish epididymo-orchitis from segmental testicular infarction; epididymo-orchitis are characterized by epididymis enlargement, increased blood flow, and enhanced echo. Moreover, CT can identify testicular torsion and torsion of the appendix testis. Typical imaging findings had increased volume and a slightly uneven increase in density of the testicle or epididymis on the affected side, and the Enhanced scan showed uneven enhancement/annular enhancement. Currently, color Doppler ultrasonography has become the first choice for testicular infarction diagnosis [4], while contrast-enhanced ultrasound can improve the accuracy of diagnosis [5]. Ultrasonography is reliable as it presents well-delineated, avascular, or markedly reduced blood flow, wedge-shaped, or round hypoechoic lesions [3]. However, round lesions or lesions with blood flow that has not completely disappeared are difficult to differentiate from hypovascular testicular tumors [6]. In such cases, magnetic resonance (MR) is more reliable than ultrasonography [2, 7]. MR findings and negative tumor markers can confirm the diagnosis and assist in the differentiation. At present, conservative treatment is the main treatment option for segmental testicular infarction. In the present case, after conservative treatment, the infarct volume was reduced, the blood flow around the infarct was increased, and blood flow signals appeared in the infarct. We then performed a correlation analysis to investigate the causes of these changes.

## Case presentation

**Fig. 1:**
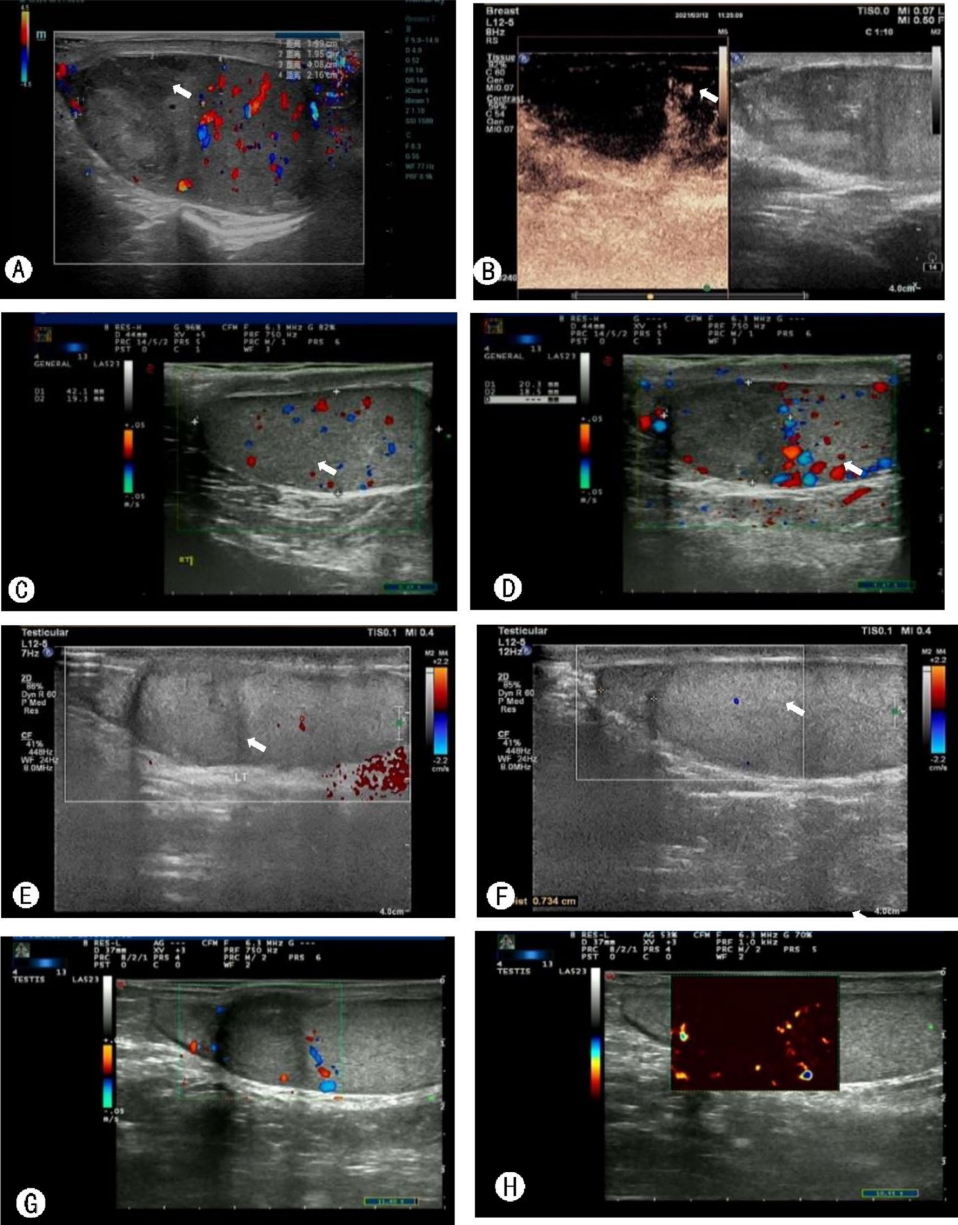
Ultrasonography was performed after infarction, revealing uneven localized heterogeneous echogenicity with no internal blood flow signal in the upper pole of the left testis, measuring 24 mm * 26 mm (**A**). The upper part of the left testicle showed an avascular area (**B**). One week after the infarction, the ultrasound showed that the echogenicity of the upper half of the left testis was reduced, measuring 20 mm * 19 mm (**C**), and several blood flow signals (**D**) were seen at its edge. Three weeks after the infarction, the ultrasound showed that the echogenicity of the upper half of the left testis was reduced, measuring 15 mm * 16 mm (**E**), and several punctate foci with strong echogenicity (**F**) were observed. Five weeks after infarction, the ultrasound revealed heterogeneous localized echogenicity in the left testis, measuring 14 mm * 15 mm (**G**), and enhanced signals (**H**) appeared in the infarct after contrast

**Fig. 2:**
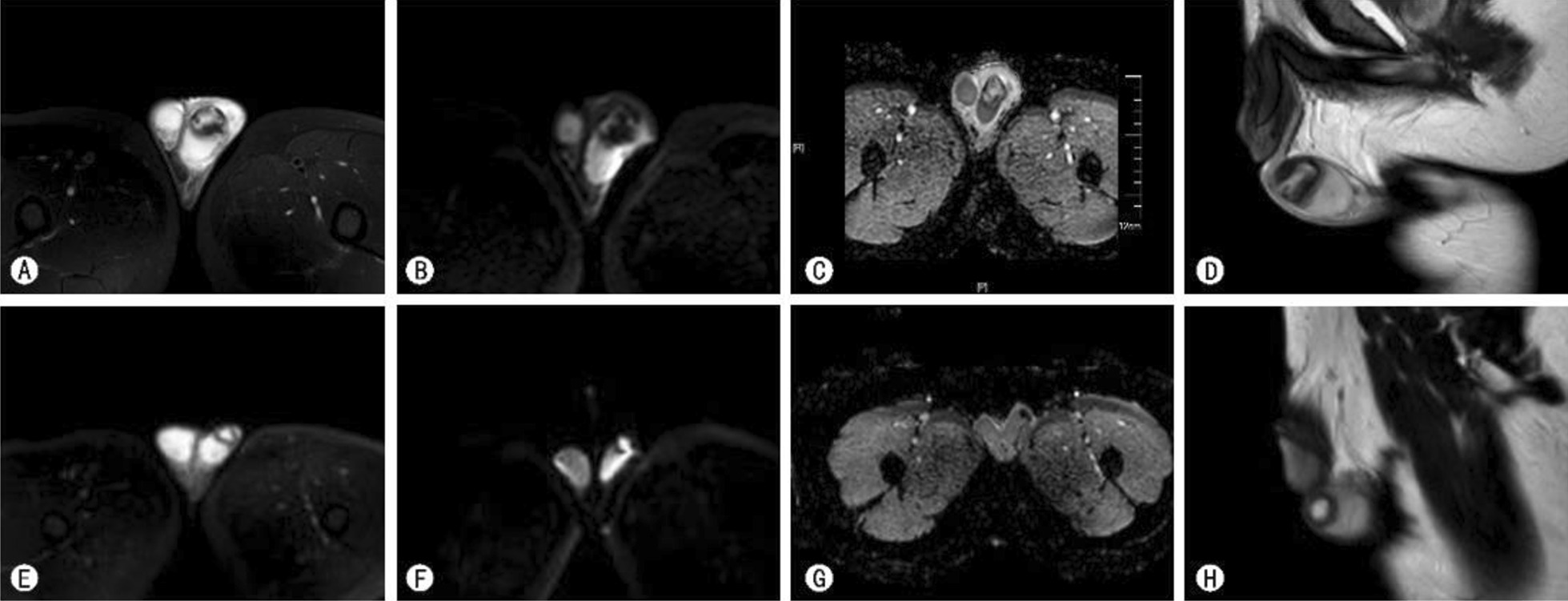
MR examination of the infarction showed a round lesion in the left testis with mixed-signal intensity (low signal intensity on T1WI, mixed high signal and low signal intensity on T2WI, low signal intensity on DWI, mixed high signal, and low signal intensity on ADC). The lesion was with clear boundary, measuring 22 mm * 19 mm, with ring enhancement at the edge, without obvious enhancement (**A**, **B**, **C**, **D**). Five weeks later, MR revealed a round lesion in the left testis (mixed high signal and low signal intensity on T1WI and T2WI, high signal intensity in the center, low signal intensity at the edge on DWI, and low signal intensity on ADC). The lesion had a clear boundary, measuring 12 mm*12 mm, with ring enhancement at the edge, without obvious enhancement (**E**, **F**, **G**, **H**)

**Fig. 3:**
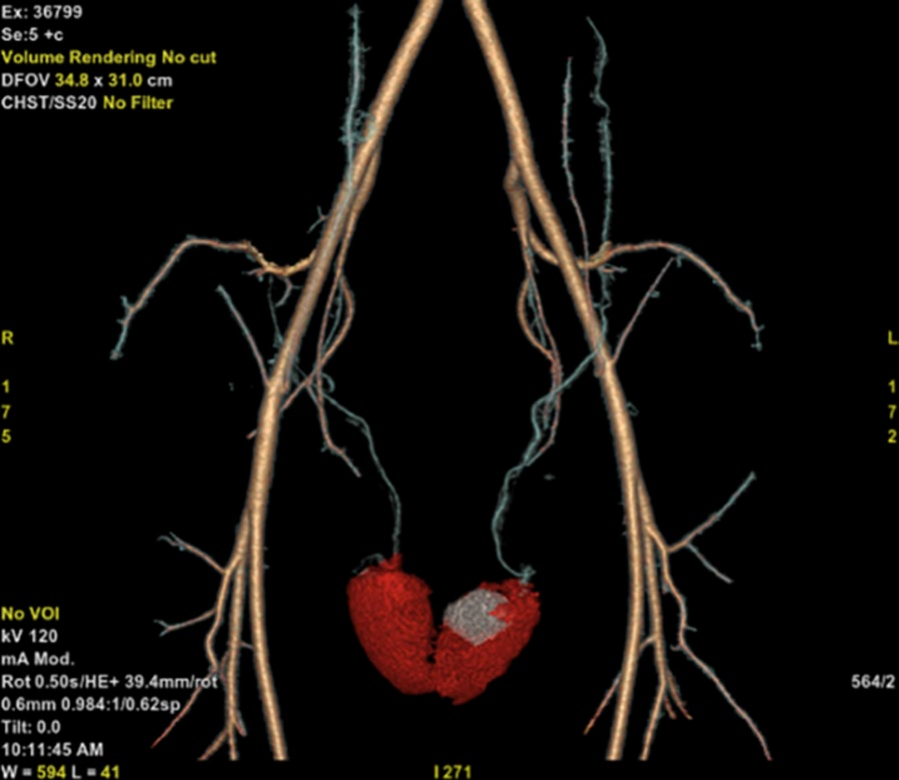
CT examination after infarction, three-dimensional reconstruction of CT showed a low-density area in the upper part of the left testis, with no obvious enhancement (Fig. 3)

The subject is a male patient, 33 years old. On March 11, 2021, he was admitted with a 5-day history of left scrotal pain and no history of chronic diseases or trauma. Physical examination showed that the upper part of the left testis was tender, hard, and swollen than the lower part. The Prehn’s sign was negative, and the right testis showed no obvious abnormality. Laboratory tests revealed normal tumor markers (AFP, HCG) and decreased testosterone. Color Doppler ultrasonography showed that the upper part of the left testis had localized heterogeneous echogenicity and an avascular area, while the other testicular areas had normal echogenicity and blood flow. Thus, the possibility of infarction was considered (Fig. 1A, B). MR of the testis showed focal necrosis of the left testis with thickening and distortion of the left spermatic vein (Fig. 2A–D). Three-dimensional reconstruction of CT showed a low-density area in the upper part of the left testis, which did not show obvious enhancement (Fig. 3). According to the patient’s physical signs and relevant results of laboratory tests, and after excluding the possibility of hypovascular testicular cancer, segmental infarction of the left testis was considered. The patient was then administered conservative treatment, including analgesia and oral testosterone undecanoate to supplement testosterone (80 mg, bid). Color Doppler ultrasound was repeated after 1 week, 3 weeks, and 5 weeks and the results showed that the infarct size was significantly reduced than before (Fig. 1C, D, E, F, G, and H). Testosterone levels returned to normal on the retest 3 weeks later. MR of the testis showed that the abnormal signal lesion in the left testis was smaller than before (Fig. 2E–H). Before the publication of this case report and any accompanying images, the patient’s written informed consent had been obtained.

## Discussion and conclusions

Segmental testicular infarction occurs mainly at the upper pole of the testis because the blood supply of the upper pole is provided by the testicular artery only, thus lacking collateral blood supply; the lower pole, however, is supplied by both the testicular artery and the vas deferens artery. Partial testicular infarctions are likely to occur when the arterial blood flow is impaired due to abnormal centripetal arteries, testicular artery branches, or anterior epididymal arteries [2]. The study by Bertolotto et al. showed that segmental infarction tends to be on the left testis, which could be related to its anatomy [5].

There are many reasons for segmental testicular infarction. According to the medical history and examination results, this case is idiopathic. Conservative treatment and follow-up are recommended if the imaging features suggest segmental testicular infarction and the tumor markers are negative. The conservative treatment of segmental testicular infarction faces some risks such as aggravation of the testicular infarction, testicular atrophy, decreased sex hormone levels, and contralateral testicular torsion, which may affect fertility in the future. The risks of surgical exploration include removal of the affected testis, postoperative bleeding, infection, unrelieved or even aggravated scrotal pain, aggravated testicular infarction, testicular atrophy, etc. Based on the results of laboratory tests and imaging examinations, conservative treatment was preferred [2] to protect the patient’s fertility.

Follow-up imaging after conservative treatment revealed that the size of the testicular infarct was significantly reduced than before. Sentilhes et al. [8] reported the reduction of testicular infarct after 3 months. The same finding was reported by Perez et al. [2], whose hypothesis was due to fibrosis of the testicular infarct focus, which was later verified by the pathological findings of the surgically removed lesion tissue; it showed that hyalinosis and fibrosis occurred in the infarcted tissue and mild contraction of the testicular tunica albuginea occurred close to the lesion.

We found that 1 week after the onset of infarction, there was an increase in blood flow around the infarct with rim enhancement, similar to Gianfrilli et al. [1] findings. In the case report by Bertolotto et al. [5], the same finding was observed in 6 of 9 patients within 2–17 days after the onset of infarction. Bilagi et al. [3] interpreted it as a mass effect; that is, edema in the infarcted area leads to displacement and compression of the surrounding normal testicular tissue so that the vessels around the infarct increase. Another explanation is that after the infarction, the hypoxia of the tissue can induce the tissue to secrete vascular endothelial growth factors that could promote the proliferation of blood vessels in the tissue around the infarct [9, 10]. This feature is also present in subacute infarction of the testis, but over time, rim enhancement gradually disappears as the infarct shrinks [5, 11].

In this case, blood flow signals appeared in the infarcted lesion 5 weeks after infarction. Consistently, in the case report of Bertolotto et al. [5], blood flow signals appeared in 12 of 14 patients 1 month after the infarction. A possible explanation is that the infarct scar is a living, dynamic tissue [12–14] with myofibroblasts and neovascularization. In cardiac pathology studies, temporary neovascularizations appear in the infarct within 7–14 days after myocardial infarction [15]. Another explanation is that the blood flow in the ischemic area of the infarct is partially restored. In 1981, Astrup et al. [16] first proposed the theory of reversible ischemic penumbra around localized cerebral infarction. According to that theory, the area around the ischemic core of the occluded artery is not immediately dead but undergoes progression from reversible ischemia to irreversible infarction. Applying the same concept in our case, the testicular tissue that has not been fully infarcted may also restore partial blood flow from a reversible ischemic state.

## Conclusions

The development of imaging has greatly improved the accuracy of diagnosis of segmental testicular infarction. This case study examined the pathological mechanisms of testicular infarcts size reduction and the increased peri-infarct blood flow after administrating conservative treatment. The causes of blood flow changes within the testicular infarcts after conservative treatment have been deduced mainly from infarcts studies in other organs such as the heart and brain. Further experimental verification of the specific underlying pathological mechanisms is needed.

## Data Availability

All authors declare that raw data and other materials are available upon requested.

## Abbreviations

MR: Magnetic resonance
AFP: Alpha-fetoprotein
HCG: Human chorionic gonadotropin
